# Resolving Myocardial Activation With Novel Omnipolar Electrograms

**DOI:** 10.1161/CIRCEP.116.004107

**Published:** 2016-07-19

**Authors:** Stéphane Massé, Karl Magtibay, Nicholas Jackson, John Asta, Marjan Kusha, Boyang Zhang, Ram Balachandran, Milica Radisic, D. Curtis Deno, Kumaraswamy Nanthakumar

**Affiliations:** From the The Hull Family Cardiac Fibrillation Management Laboratory and University Health Network, Toronto General Hospital, Toronto, ON, Canada (S.M., K.M., N.J., J.A., M.K., K.N.); St. Jude Medical, St. Paul, MN (R.B., D.C.D.); and Institute of Biomaterials and Biomedical Engineering, University of Toronto, Toronto, ON, Canada (B.Z., M.R.).

**Keywords:** cardiac arrhythmia, cardiac electrophysiology, catheter ablation, conduction velocity, omnipole

## Abstract

**Background—:**

With its inherent limitations, determining local activation times has been the basis of cardiac mapping for over a century. Here, we introduce omnipolar electrograms that originate from the natural direction of a travelling wave and from which instantaneous conduction velocity amplitude and direction can be computed at any single location without first determining activation times. We sought to validate omnipole-derived conduction velocities and explore potential application for localization of sources of arrhythmias.

**Methods and Results—:**

Electrograms from omnipolar mapping were derived and validated using 4 separate models and 2 independent signal acquisition methodologies. We used both electric signals and optical signals collected from monolayer cell preparations, 3-dimensional constructs built with cardiomyocytes derived from human embryonic stem cells, simultaneous optical and electric mapping of rabbit hearts, and in vivo pig electrophysiology studies. Conduction velocities calculated from omnipolar electrograms were compared with wavefront propagation from optical and electric-mapping studies with a traditional local activation time–based method. Bland–Altman analysis revealed that omnipolar measurements on optical data were in agreement with local activation time methods for wavefront direction and velocity within 25 cm/s and 30°, respectively. Similar agreement was also found on electric data. Furthermore, mathematical operations, such as curl and divergence, were applied to omnipole-derived velocity vector fields to locate rotational and focal sources, respectively.

**Conclusions—:**

Electrode orientation–independent cardiac wavefront trajectory and speed at a single location for each cardiac activation can be determined accurately with omnipolar electrograms. Omnipole-derived vector fields, when combined with mathematical transforms may aid in real-time detection of cardiac activation sources.

## Abstract 2

Supplemental Digital Content is available in the text.

**WHAT IS KNOWN**

- With catheter mapping, unipolar recordings often have far field artifacts and bipolar recordings have directional dependencies and complex shapes that prevent single point catheter-based assessments of conduction velocity and activation direction.
- Current activation maps require time-consuming collection and temporal correction from multiple sites.

**WHAT THE STUDY ADDS**

- We introduce an Omnipolar Technology (OT) method implemented in a 3D electroanatomic mapping system that derives the direction of wave front activation from a single point and is independent of catheter orientation.
- When combined with high-density mapping catheters, OT may enable a “move and locate” strategy to potentially identify the arrhythmia focus without mapping the entire chamber.
- It also offers the possibility for improving electrogram characterization of myocardial arrhythmogenic substrate.

The identification of cardiac arrhythmia sources today requires accurate activation time assessment of propagating wavefronts. For almost a century, since Sir Thomas Lewis introduced isochronal activation maps, there has been no basic advancement to this concept.^1^ Wavefront speed and direction can be challenging to determine with traditional bipolar methods because ambiguity arises from the orientation and placement of electrodes on the area of interest. Furthermore, traditional mapping techniques rely on precise timing and electrode positions to accurately determine the velocity of a propagating wave.^2,3^ Strategies have been proposed to deal with the above challenges by incorporating multiple local assessments and deriving vector patterns of activation.^4–9^ These techniques are inherently limited in accurately determining the local activation times (LATs) because of inadequate sampling and imprecise times and positions to provide a wavefront’s velocity^10^ and hence create a need for alternative mapping strategies. An omnipole that resolves signals from all possible simultaneous bipoles (from every direction around the mapping location) from the electric field generated by a travelling wave could provide a definitive assessment of local cardiac wavefront properties, such as speed and direction.

The paradigm of instantaneously measuring cardiac wavefront properties at a single location has not been explored. If proven and tested, this could save time and provide useful information without the tedious creation of isochronal maps. The ability to accurately determine and display beat-to-beat activation direction (AD) and conduction velocity (CV) without the need for global mapping would enhance the efficiency of cardiac mapping and treatment of cardiac arrhythmias. This is of particular value if performed with an ablation catheter without acquiring and editing LATs.

In this article, we provide the basis for an orientation- and LAT-independent omnipolar-mapping approach to characterize beat-by-beat cardiac activation and validate against mapping standards.^11–13^ To do so, we applied the biophysical basis of omnipolar electrograms, methodically, starting on a cardiac monolayer, followed by 3-dimensional (3D) constructs built with cardiomyocytes derived from human embryonic stem cells and then on whole Langendorff perfused rabbit heart. Finally, we tested the reproducibility and consistency in controlled conditions and then tested it in vivo conditions in porcine electrophysiology studies. We then used the omnipolar concept in concert with mathematical transforms of divergence and curl^14^ to objectively reveal cardiac sources in a mapping chamber.

## Methods

### Experimental Models and Data Acquisition

Validation of the technique was performed using propagation of wavefronts during optical and electric mapping in preclinical electrophysiology studies. Data were acquired from various types of biological media, such as monolayer cell preparations, human induced pluripotent stem cell derived 3-dimensional cardiac constructs, isolated whole rabbit hearts and in vivo swine electrophysiology studies. The technique was validated against established CV methods that use both electric and optical LAT-based isochrone maps. Reproducibility was then tested in vivo in porcine electrophysiology studies, which were approved by an Institutional Animal Care and Use Committee, and the studies conformed to the guide for the care and use of laboratory animals (APS Protocol No. CER233). Rabbit experiments in this study were performed under an Animal Use Protocols approved by the Animal Care and Ethics committee at the Toronto General Hospital.

### Neonatal Mice Cardiomyocyte Monolayer Preparations

Cardiac monolayer cells were prepared after the procedures published previously^15^ and presented in detail in Methods section in the Data Supplement. Overall, 8 monolayer plates were optically mapped with a complementary metal-oxyde semiconductor (CMOS) camera (MiCAM Ultima, SciMedia, Costa Mesa, CA) with a spatial resolution of 0.05 mm/pixel, sampled at 333 Hz. In addition, 9 plates were electrically mapped from which unipolar signals were collected, sampled at 5 kHz, and band-passed filtered between 1 Hz and 1 kHz, using a microelectrode array or MEA (Multi Channel Systems MCS GmBH, Reutlingen, Germany).

### Three-Dimensional Myocardial Constructs Derived From Induced Pluripotent Human Stem Cells

Three myocardial tissue constructs were created for this study from human embryonic stem cells–derived cardiomyocytes, according to the following procedure^16–20^ described in detail in the Data Supplement. With the myocardial tissue filling the scaffold pores, the cells were incubated with a calcium-sensitive dye, Fluo-4 (ThermoFisher Scientific, Waltham, MA), for 20 minutes and subsequently the constructs were optically mapped with the same set up as described above.

### Whole Rabbit Heart Langendorff-Concurrent Langendorff Optical and Electric Rabbit Data

The purpose of this model was to validate the electric mapping acquired unipoles, bipoles, and omnipoles against concurrently obtained optical-mapping data. Whole hearts were harvested from 6 healthy New Zealand White male rabbits weighing ≈3 to 4 kg. After a heart was explanted, it was placed in a cold Tyrode solution, which was then delivered to an adjacent room, <5 minutes away. The heart was flushed thoroughly to remove any blood particles. The heart was cannulated through the aorta and was secured with 2-0 silk sutures to the cannula. The heart was perfused with Tyrode solution (95% O_2_ and 5% CO_2_) at a variable flow rate, with its pressure and temperature maintained at ≈55 mm Hg and 37°C, respectively. An 18-pole electrode array (St. Jude Medical, EnSite HD Grid catheter, St. Paul, MN) was stitched to the left ventricular free wall, and pacing electrodes were placed on predetermined sites to create different controlled wave propagation on the epicardium. The heart was allowed to stabilize for about 10 minutes after which our mapping protocol was executed. Concurrently, unipolar and bipolar signals were acquired from the 16 electrodes forming a 1.2×1.2 cm^2^ of the EnSite HD Grid catheter using a recording system described before (UHN Cartesian Cathlab Mapping System).^21^ Simultaneous unipolar and bipolar electrograms were recorded while optical images were obtained as described in the Methods section in the Data Supplement. For these optical experiments, a voltage dye (DI-4-ANBDQPQ) was used.

### In Vivo Porcine Electrophysiology Study

The objective of this preclinical component was to compare the accuracy and repeatability of omnipolar electrograms compared with bipolar electrograms while assessing commonly used electrophysiology parameters, such as activation timing and CV. For this study, a research version of the EnSight Velocity cardiac mapping system with real-time, beat-to-beat omnipolar processing was used.

Four 45±5 kg Yorkshire cross swine were used for this study. Each animal was sedated using intramuscular injection of narcotic and then anesthetized with isofluorane. After applying Ensight NavX and defibrillator pads, vascular access was established for neck and femoral vessels and catheters were deployed. Two catheters were used to test omnipolar electrograms (EnSite HD Grid catheter), which has 18 electrodes disposed over 4 splines, 16 of them making a 2-dimensional square 4×4 grid and the S120, an ablation catheter specially made for omnipolar analysis that features a 3-electrode ring configuration next to its distal electrode, thereby forming a tetrahedron pointing distally. A second tetrahedron is also found on the proximal side of the ring configuration. Omnipoles were generated on EnSite HD Grid catheter using the 2D method described below. These catheters were deployed successively in 3 predetermined locations: A and B in RA and C in LA. Three site pair positions (AB, BC, and CA) were used to assure both catheters reached all positions. Three rhythms for each pair were created by pacing different electrode pairs from a third catheter inserted into the coronary sinus. These 3 stimulation modalities in addition to normal sinus rhythm made up for a database of various ADs and speeds seen by the 2 omnipolar catheters. For each condition, 20 s of electrograms were acquired by the EnSite Velocity system, for a total of 72 recordings available for analysis. Twenty-four of them were randomly selected for analysis and chosen as to make a set comprising all possible combinations of stimulation modalities and electrode locations. Electrode spatial coordinates and corresponding electrograms were exported and analyzed in custom software developed in Matlab (Mathworks Inc) and previously validated.^11^

### Signal Processing for Omnipolar Electrograms

**Figure 1.**
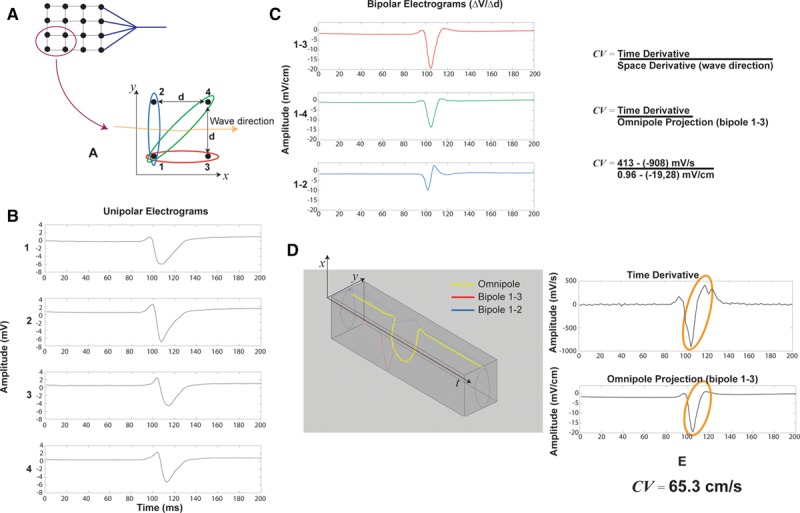
Biophysical basis of omnipolar mapping. **A**, **Top left**, A 2-dimensional electrode array (EnSite HD Grid catheter) with a group of electrode selected for analysis. **Bottom right**, An electrode clique made of 4 electrodes here disposed in a square of side *d.* A coordinate system (*x* and *y*) is shown along with an arrow depicting the direction of a travelling wave crossing the clique. Three bipolar electrode arrangements are also shown. This arrangement can be repeated on the electrode array by using consecutive electrodes. **B**, Unipolar electrograms recorded from the 4 electrodes shown in **A**. **C**, Bipolar electrograms derived from the bipolar electrode arrangement is seen in **A**. Note that the vertical scale includes the spatial distance *d* (0.4 cm) or 1.4d (0.56 cm). **D**, Omnipole concept showing the signal obtained by evaluating the E-field generating the bipoles is shown in **C** (in yellow). Also shown is 2 of the omnipole projections (bipole 1–3 in red and 1–2 in blue) demonstrating the relationship between the omnipole and commonly used bipolar electrograms. **E**, Calculation of conduction velocity (CV) using the ratio of time over spatial derivative. Here, the omnipole is searched to identify the direction along which the time and spatial derivative signals match. This in turn provides the propagation direction, in this example the *x* axis. Velocity amplitude is then computed by using the ratio of the peak-to-peak values from both the signals.

In practice, local unipolar signals are used to derive speed and activation and are obtained from a group of 3 to 4 closely spaced electrodes called a clique. This clique could be from an array or it could be from a single catheter with closely and differentially spaced electrodes. The CV vector and other omnipolar signal characteristics are considered to be located at the center of these cliques. An example of a 4-electrode clique is shown in Figure 1A. For simplicity, we will assume that these 4 electrodes are laying on a flat myocardial surface. Here, we also define a 2D coordinate system (*x* and *y* axes) and show distance (d) between the electrodes. For simplicity, the clique in this example forms a square, and a wave is traversing the clique generally from left to right along the *x* axis. Figure 1B shows the unipolar electrograms recorded from these 4 electrodes, labeled as 1, 2, 3, and 4. At this point, these local electrograms could have been recorded by any electrophysiology recording system. Commonly, we apply a space derivative to these signals to better reveal local information—we have called this derivative bipolar electrograms. Three bipolar electrograms are shown in Figure 1C. Not surprisingly, the largest amplitude is shown on bipolar 1 to 3 (same axis as the travelling wave), whereas the bipole perpendicular to the wave is the smallest. This spatial derivative can be expressed mathematically as shown below:

Let 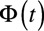 be a collection of unipolar electrograms (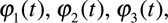
and 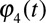) from a 4-electrode clique organized as follows:


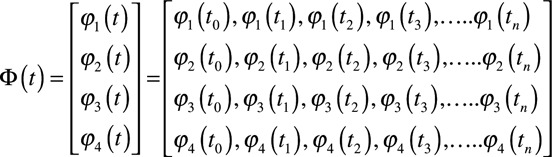


where n is the number of samples taken at different time points (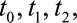 etc.).

We also have the following bipoles:


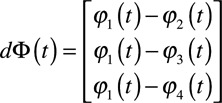


shown in Figure 1C and a distance matrix: 
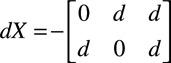


where each column contains the (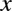 and 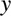) distance for each bipole in 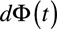. For simplicity, we will assume that the electrode coordinates do not change over time; therefore, the distance matrix will be a constant.

We also know that unipolar electrograms are related to an electric field at the extracellular–myocardial interface. The relationship between this field 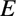 and unipolar voltages is well known:


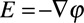
(1)

The relationship between 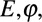 and 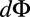 is described in details in a separate technical article.^11^ This article introduces the omnipole concept, a least square solution for 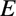.

This concept is illustrated in Figure 1D. Here, the omnipole (thick yellow tracing) is shown with its *x* and *y* components as a function of time, along with 2 of its traditional bipolar projections (namely 1–3 and 1–2 from Figure 1C). We call this signal an omnipole because it is independent of electrode orientation. On the edge of the 3D projection, we can see the loop made by the omnipole during the wave propagation.

Recalling that the omnipole in this example is produced by a travelling wave with a propagation direction almost parallel to the *x* axis, we can also demonstrate that (1) for an observer travelling with the wave, the change of voltage should be zero and (2) changes of voltage perpendicular to the wave propagation should also be zero, providing that the wave is planar. This should be valid if *d*, the interelectrode distance, is small enough. The travelling wave concept and derivations are described in more details in the study by Deno et al.^11^ In our example, in a 2D context and with a wave travelling along the *x* axis we obtain:


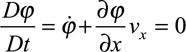
(2)

where 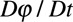 is the total time derivative (equal to 0 for observer travelling with the wave); 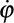, the partial time derivative of 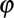; 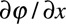, the space derivative along the wave propagation; and 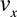, the velocity of the travelling wave.

Simplifying Equation (2) we obtain


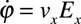
(3)

Where 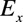 is the component of *E* orientated along the direction of the travelling wave, in our example, along the *x* axis.

A CV vector (***v***) consists of a magnitude (CV) and an activation direction (AD). At a fixed location, as the traveling wave passes by, CV can be found from (Equation 3) using the concept of phase velocity as derived to be the ratio of temporal and spatial derivatives of 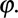



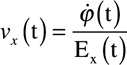
(4)

This concept has been presented recently.^11,12,22^ Assuming the wavefront to locally propagate with a nearly constant CV, the 2 signals, 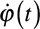 and 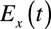, must resemble each other. The term omnipole finds its origin in the search of a spatial derivative in every possible direction around the clique of electrodes to maximize correlation to the temporal derivative. Thus, the omnipolarity of the search yields the wavefront direction. Their ratio is a multiplicative constant that describes a wave’s CV. This fact allows us to choose for each beat’s wavefront an AD (in this example, 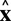) to maximize the correlation of 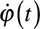 and 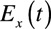. Figure 1E provides a visual representation of how the CV of the propagating wave is calculated. In this case, the closest match to the time derivative is a close approximation of bipole 1 to 3 as the wave is propagating along the *x* axis.

The acquisition and processing of omnipolar electrograms from cliques to calculate omnipolar electrogram signals 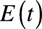, AD, and CV, are repeated throughout the field of view within a selected media using consecutive electrode cliques to obtain a vector field from a set of adjacent velocity vectors, **V**. It should be noted that because this concept applies to any traveling wave, omnipolar signal processing could also be applied to fluorescence signals obtained using voltage or calcium fluorophores.

### Validation of CV Measurements: Rabbit Langendorff Model

Validation of CV measurements was performed in 2 steps. In the first step, optical signals were used to compare measured CVs from omnipolar electrograms with CVs calculated from high-resolution isochrone maps using established activation time methods.^4^ Optical data were analyzed as follows: gold standard optical isochrone maps were generated using custom written software in IDL 7.1 (Exelis Visual Information Solutions, Boulder, CO), using the maximum positive deflection of each optical signal. A vector field was created from a 10×10 grid of recording sites. For each recording site, the average activation value of a 7×7 pixel grid was used and then CV was calculated using a similar method used by Bayly et al^4^ and described in the Methods section in the Data Supplement. Omnipolar electrograms and vectors were computed as above from the same 10×10 grid and compared with the gold standard (speed and direction).

In step 2, the same gold standard was used to match electric data. Nine CV vectors were calculated from the 16 electrodes of the EnSite HD Grid catheter using omnipolar electrograms. These vectors were compared with optical determinations made at recording sites chosen such that their central point within each clique was aligned with the central point of the equivalent electric clique. Epicardial waves were created by electrically stimulating the heart at 4 predetermined sites as described in Methods section in the Data Supplement. Cliques where a planar wave could be identified on the corresponding optical isochrone maps were used for the analysis. All omnipolar processing was performed offline with Matlab.

### Accuracy and Repeatability of Omnipolar Electrograms Versus Bipolar Electrograms: In Vivo Swine Model

The principal aim of the in vivo study was to demonstrate the beat-by-beat use and consistency of omnipolar electrograms for its near-clinical application. For each recording and for each beat, unipolar, bipolar, and omnipolar electrograms were measured to further compare traditional practices with the novel omnipolar algorithm. An analysis was performed to evaluate the consistency of velocity vectors obtained from omnipolar electrograms in terms of magnitude and direction performed in Matlab.

### Divergence and Curl

We used these mathematical operators on a vector field of AD generated by the omnipolar algorithm to quantify the location and orientation of sources within a myocardial surface. Divergence and curl have been used previously to characterize cardiac rhythm features in animal and human models, such as focal sources, pacing, and arrhythmic sites.^13,23–25^ In this study, we apply divergence and curl to a vector field derived from the omnipolar algorithm, which does not rely on isochronal maps. Briefly from the vector field generated using the AD vectors, one may locate sources and quantify the degree to which they resemble foci or possess rotation using divergence and curl, respectively. Sources, as described by a vector field radiating outward, and sinks as described by converging inward can be mapped by calculating the divergence of the field. In addition, sources can be described to have some vorticity quantified by Curl. Such vector field features could be used to characterize focal, pacing, and arrhythmic sources as described in the Methods section in the Data Supplement.^24^

### Statistical Analysis

Ex vivo CV was validated using Bland–Altman analysis to compare magnitude and direction between each independent and paired measurement. Agreement limits between the 2 methods were defined as 95% confidence interval (95% confidence interval=mean difference±1.96 SD). We have set the acceptable clinical limit for this interval to be ±25 cm/s for the velocity and ±30° for the direction of the travelling wave. Results in this section are presented as mean difference (or bias) ±1.96 SD. Statistical analysis for Bland–Altman was performed using Prism 5 (GraphPad Software, La Jolla, CA).

In vivo comparison of beat-to-beat CV amplitude was conducted by using a general linear model, incorporating random effects for subject, rhythm, site, and clique. The category of measurement (eg, omnipolar technology, bipolar, or unipolar) was modeled as a fixed effect, and *P* value of <0.05 was considered significant. Tests comparing SDs were conducted from pooled data using Levene test for equal variance. This analysis was performed using Minitab 17 (Minitab, State College, PA).

## Results

### Omnipole-Derived Vector Fields

#### Neonatal Mice Cardiomyocyte Monolayer

**Figure 2.**
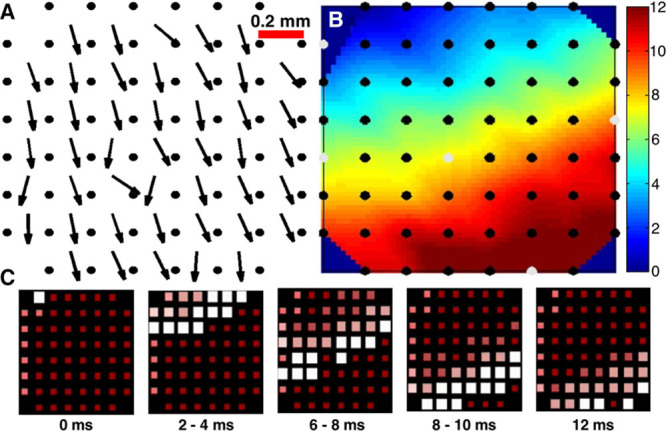
A vector field derived from omnipolar electrograms of electrically mapped cardiac monolayers illustrates the application omnipolar mapping; the field in **A** depicts a uniform wave propagation from the **top left** corner of the MEA field toward the bottom right. As a reference, we verified the general direction of the field by deriving its corresponding isochronal map (**B**) and comparing it with action flashing dot display (**C**) to show the wave propagation in time that were found to be in agreement with each other.

Here, we evaluated the ability of omnipolar electrograms to demonstrate wavefront direction compared with traditional isochronal mapping. In all 9 electrically mapped and 8 optically mapped preparations, the vector field generated by omnipoles, for both modalities, described well the direction of activation within cardiomyocyte monolayers. An example of an electrically mapped monolayer is shown in Figure 2A. Using vectors generated from omnipolar electrograms, we determined a downward-oriented vector for each clique of electrodes within the MEA field, depicting largely a uniform propagation, with the general direction of activation from the top left to the bottom right of the MEA field, as verified with its LAT (Figure 2B) and associated flashing dot sequence (Figure 2C; Movie I in the Data Supplement).

**Figure 3.**
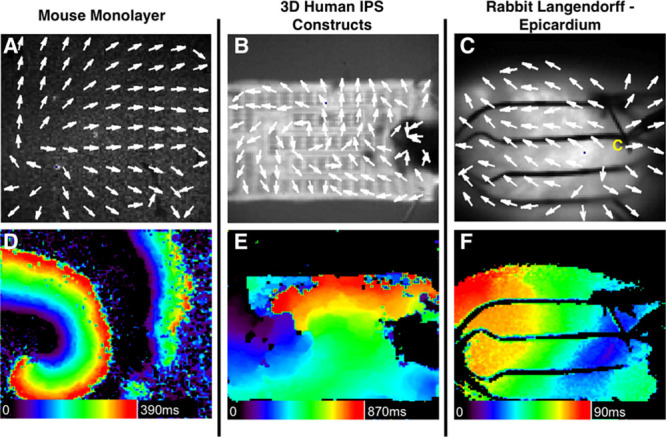
To examine the versatility of omnipolar mapping, we show here the omnipolar-derived vector fields from optically mapped cardiac monolayer (**A**), human induced pluripotent stem cell (IPS) myocardial construct (**B**), and a rabbit heart (**C**) showing the left ventricle with apex on left and base on right under electric stimulation (shown in picture as site “c”). As with Figure 2, we verified the general direction of each vector fields by obtaining the corresponding local activation times maps for cardiac monolayer (**D**), construct (**E**), and rabbit heart (**F**). 3D indicates 3-dimension.

The above propagation described a linear wavefront within a monolayer. In addition, 8 complex propagations from optically mapped monolayers exhibiting stable rotors were studied. Omnipole-derived vector fields for each plate demonstrated activation patterns of the wavefront as illustrated in Figure 3A (Movie II in the Data Supplement). Collectively, the vectors in Figure 3A show a clockwise rotor with a rotor core located at the lower left that gradually turns to uniform centripetal propagation as one moves away from the rotor core. Vector field orientation was verified by deriving its corresponding LAT map as shown in Figure 3D.

#### Three-Dimensional Human Induced Pluripotent Stem Cell Myocardial Constructs

A monolayer is a simple example of excitable media; although cardiomyocytes can form lateral junctions (in *x*–*y* plane) in the monolayer, they are not connected to other cells in a 3-dimensional arrangement as in the native heart. As such, we tested the use of omnipolar electrograms in 3D constructs that were conditioned to exhibit lower than the normal CV to allow re-entry and formation of rotors. Shown in Figure 3B is an example of the use of omnipolar electrograms on one of the 3D myocardial constructs, each of which exhibited a rotor (Movie III in the Data Supplement). The versatility of omnipolar electrograms in characterizing local wavefront directions by applying the technique in a realistic 3D construct was evaluated. Similar to observations with optically mapped monolayers, a group of omnipole-derived vectors was seen to form around a rotor core seen at the left side of the construct shown in Figure 3B. Omnipole-derived vectors were able to characterize the existing rotors from all the 3 construct preparations at 7 different time points. The omnipole-derived vectors also provide direction of rotation (counter clockwise, as seen in Figure 3B) of the rotor about its core, with vectors depicting uniform wave propagation at the sides of the 3D construct as they move further away from the core. We used LAT maps to validate the results we obtained from omnipolar electrograms, an example of which is shown in Figure 3E showing a similar rotational pattern about the rotor core.

#### Whole Heart Rabbit Langendorff

In the previous sections, we have characterized the application of omnipolar electrograms in the case of uniform wave propagation and its characterization of rotating sources. Using our rabbit heart Langendorff data, we studied propagation of waves from focal sources. Shown in Figure 3C is an optically mapped epicardium of a rabbit heart (Movie IV in the Data Supplement), also showing the placement of the EnSite HD Grid catheter and pacing electrode “C” from our unique concurrent electric-optical mapping setup. The optical data, with omnipole-derived vector field, indicated the existence of a focal source and its approximate location on the epicardium. A group of vectors in a star burst pattern diverging from the location of the pacing electrode identifies the source of activation. As we move away from the source, we observe more uniform wave propagation toward the apex of the rabbit heart. Similar observations were made from all of the pacing sites for each rabbit heart. Such an AD was verified using an LAT map, as shown in Figure 3F, where a wave traversing from the base of the heart (where the pacing electrode is located) travels toward its apex.

### Validation of Omnipole-Derived Vectors: Concurrent Langendorff Optical and Electric Rabbit Data

**Figure 4.**
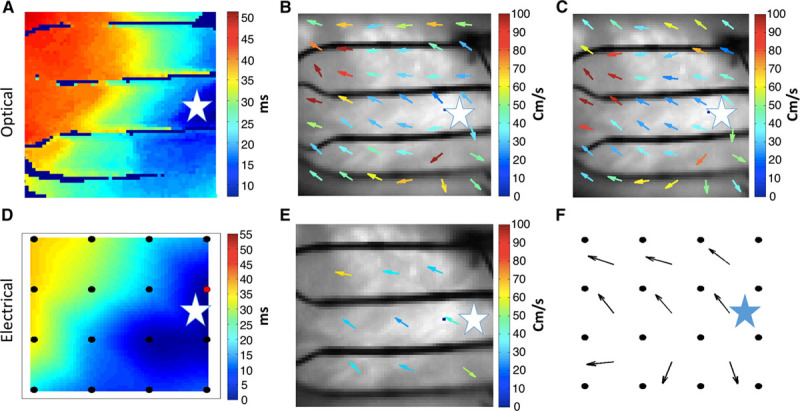
Comparison of vector fields derived by local activation times (LAT) method vs omnipolar mapping; we evaluated the similarity of vector fields calculated from a standard LAT method to those calculated from omnipolar electrograms from both electrically and optically mapped rabbit hearts. The orientation here is the same as in Figure 3C and the stimulation site (c) is represented with a white star. The overall direction of the vectors obtained from LAT method confirms the results of omnipolar mapping with high similarity values (Figure I in the Data Supplement). **A**, Isochrone map derived from optical mapping (gold standard); **B**, 6×7 velocity vector field computed from the optical isochrone map. **C**, Velocity vector field obtained from optical signals using omnipolar mapping. **D**, Isochrone map derived from electric mapping. **E**, 3×3 velocity vector field computed from the gold standard. **F**, Velocity vector field obtained from electric signals using omnipolar mapping. The star on each panel indicates pacing location.

Concurrent mapping with optical and electric signals allows for the strongest validation because the omnipolar electrograms and CV are computed in the middle of electrode cliques, and these exact locations can be evaluated by optical transmembrane potential mapping. Figure 4A shows a gold standard optical isochrone map obtained while stimulating from site “C” (represented in all panels as a white star). Figure 4B and 4C shows consistent vector fields derived from optical omnipolar signal and LAT-based method. Vector color represents conduction speed. The vector fields from separate paced beats have similar CV vectors, both in terms of direction and speed. Differences observed at the edge of the optical view field can be attributed to lower optical signal quality because of edge effects from the heart. Figure 4D shows an isochrone map based on electrogram LATs. Figure 4E and 4F shows consistent vector fields derived from electric omnipolar signal and LAT-based method from the gold standard.

Optical signals from 224 sites were used for a Bland–Altman analysis comparing conduction speed and AD using both omnipolar and traditional LAT-based methods (Figure IA and IB in the Data Supplement). Bland–Altman analysis showed that the bias between both methods was small with the confidence interval for optical CV speed at 3.9±18.5 cm/s and for optical AD being 0.6±29.5°.

Similarly, electric signals from 38 sites were used to compare conduction speed and AD using both omnipolar and traditional LAT-based methods (Figure IC and ID in the Data Supplement). Again, Bland–Altman analysis found the bias between methods to be small with the confidence interval for electric CV speed being 6.2±24.4 cm/s and for electric AD being 0.9±29.7°. Together, these results suggest that omnipolar methods can perform well in single beats to determine wave velocity and direction without the need of global mapping.

### Omnipolar Electrograms From Array Catheter Versus Single Catheter

**Figure 5.**
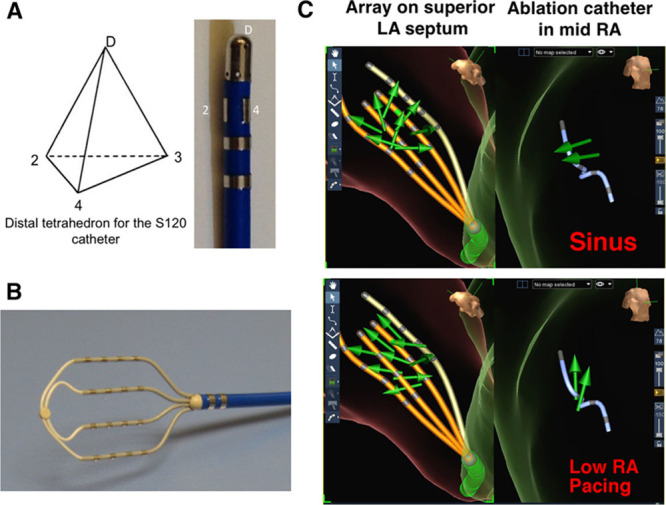
Use of omnipolar mapping with a single ablation catheter vs a 2-dimensional array catheter; shown in **A** is a segmented catheter S120: the ring beside the distal electrode is segmented to provide a 4-electrode tetrahedron pointing distally. Another tetrahedron is also obtained on the proximal side using a proximally adjacent electrode. **B**, EnSite HD Grid catheter that contains a 4×4 regular electrode grid from which 9 conduction velocity vectors can be computed. In **C**, we illustrate omnipolar mapping during a live study, whereas mapping a sinus rhythm recorded with the EnSite HD Grid catheter on superior LA septum and S120 located in mid RA (**top**). Vector fields from same arrays in same location during low RA pacing illustrating the change of conduction direction (**bottom**). LA indicates left ventricle; and RA, right atria.

With the use of the diagnostic array catheter (EnSite HD Grid) shown in Figure 5, multiple determinations of activation direction (AD) and CV can be obtained, providing confidence in the measurement made. In addition, wavefront physiology can be mapped over the surface of the array. It is important to note that these measurements can also be made on a segmented single catheter (S120), which is a mapping/ablating catheter, with a segmented group of 3 electrodes disposed at 120° and forming a tetrahedron with the distal electrode. This catheter allows 3D depiction of AD from which 3D omnipole can be derived. This will be described more in details in a future study. Movie 5 in the Data Supplement demonstrates change in CV from sinus rhythm to pacing from the coronary sinus. Single catheter determination of myocardial properties highlights here the lack of need for complicated delivery tools for complex array and ability to navigate with mapping information and ablate.

### Locate While Mapping Potential of Omnipolar Electrograms

**Figure 6.**
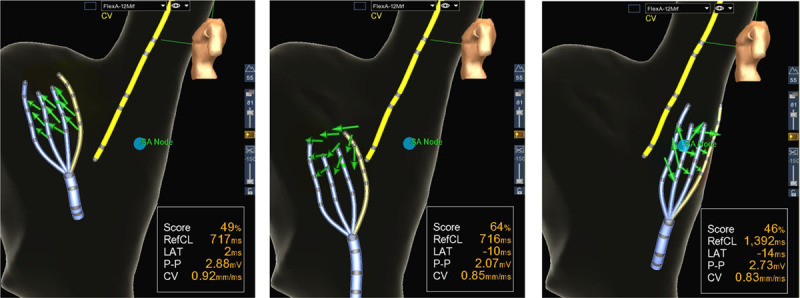
Potential clinical applications of omnipolar mapping: an illustration of the drag-and-map capability of omnipolar mapping used in porcine model. This reflects the compactness of the algorithms used for omnipolar mapping that we can perform real-time computations of vector fields to map areas of a heart. This allows for manipulating the catheter toward the activation source as opposed to mapping an entire chamber and then localizing the source. CV indicates conduction velocity; and LAT, local activation times.

Movie 6 in the Data Supplement and Figure 6 demonstrate a potential application of omnipole-derived vector fields, which allows for mapping wavefronts in real time as an omnipolar catheter is moved. Here with the EnSite HD Grid catheter array, we demonstrate a potential advance in cardiac mapping by not having to annotate electrograms and create activation maps. Traditionally, mapping requires tedious annotation and even with rapid automated annotation, it has to be completed before locating source of region of interest. A real-time depiction of AD with omnipolar electrograms obviates the need for manual annotation, hence significantly shortening the time needed to create activation maps for a whole cardiac chamber.

### Consistency and Repeatability of Omnipoles in Preclinical Swine Model

An evaluation of omnipolar performance was also sought in a more realistic, clinical context. The previous sections have validated omnipolar electrograms for determining CV and AD using cell cultures, tissue constructs, and isolated heart models with optical and electric mapping methods. Whole animal work requires a 3D electroanatomic mapping system subject to clinically relevant influences. In the absence of an optical-mapping gold standard, we sought to compare omnipole with traditional unipole- and bipole-derived CV and AD. Recognizing that these parameters would vary with location, rhythm, and subject, we quantified beat-to-beat variations with these factors fixed. A practical clinical assessment tool should provide consistent measurements from individual beats so as to allow for rapid mapping and correct interpretation of rhythms.

### Reproducibility of CV Measurements

**Figure 7.**
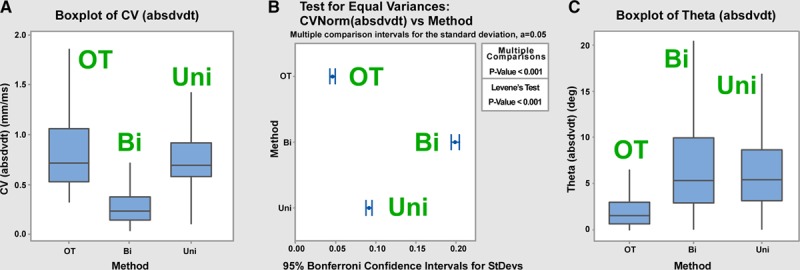
Reproducibility of omnipolar mapping from near-clinical applications. In **A**, we show the distribution of conduction velocity (CV) speed obtained from omnipolar (OT), unipolar (uni), and bipolar (bi) electrogram techniques. Whereas uni and OT give similar results, bipolar local activation times seems to underestimate CV. The variation in beat-to-beat determinations is seen in **B** to be least for OT and greatest for bi. In **C**, we see that OT direction vectors are clustered more tightly than uni or bi vectors. These findings support the use of omnipolar electrograms (Ea) as a tool to assess conduction direction. Boxplots in **A** and **C** are shown as median±interquartile range.

Mean±SD CV values encompassing all subjects, sites, rhythms, cliques, and beats were compared as shown in Figure 7A. Although both omnipolar technology and unipolar methods produced results in the physiological range (93±57 versus 70±36 cm/s, respectively), bipolar LAT methods tended to produce low CV (35±36 cm/s, *P*<0.01). Beat-to-beat temporal variation in CV was lowest when obtained from omnipolar electrograms as seen in Figure 7B. Variation expressed as a SD was distinct for all the 3 methods omnipolar technology, unipolar, and bipolar as 4.6, 9.1, and 20.3 cm/s, respectively (*P*<0.001). Omnipolar measurements of CV were more consistent because of the better use of signals and catheter orientation independence (omnipolar and local features).

The directions of activation when similarly encompassing all subjects, sites, rhythms, cliques, and beats were widely dispersed as a result of differences in the first 4 such factors. The directions of activation were also more consistent when measured with omnipolar technology with a mean angle dispersion of 1.5° versus 5.3° for unipolar and 5.4° for bipolar (Figure 7C). Seventy-five percent of omnipolar measured conduction directions were within 3.5° of the mean, suggesting a superior discrimination of subtle shifts in direction that might occur with ectopy or functional blocks.

During less well-controlled in vivo situations, additional variability and artifact were apparent. Ventilation-associated artifacts influenced omnipolar and traditional method determinations of CV and AD. Beat-to-beat angular spreads in EnSite HD Grid catheter cliques of ≤20° were common and, as anticipated, starburst spread (90–180°) was observed near sources.

### Application of Omnipoles to Detect CV Slowing

**Figure 8.**
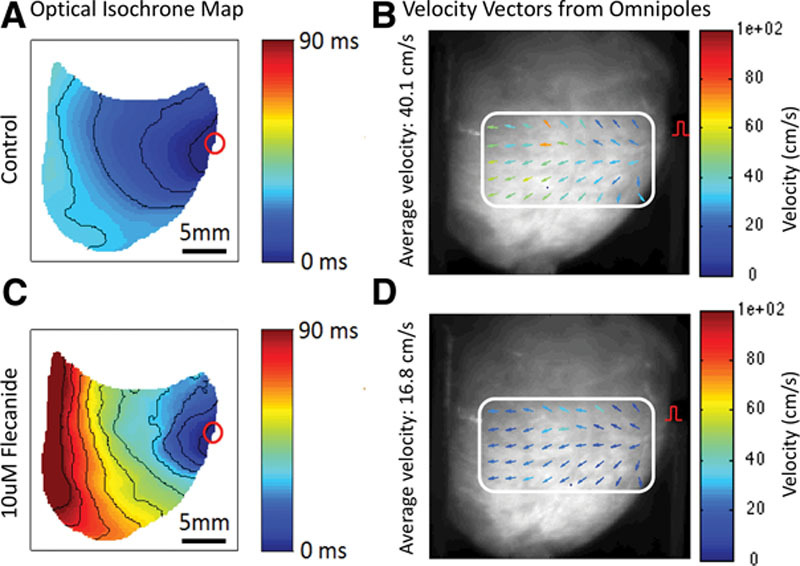
Effect of flecainide on velocity vector field. **A**. Isochrone map obtained under control conditions showing activation spreading during programmed electric stimulation (4 Hz S1, site shown as a red circle). Total activation time was 35 ms. **B**, Velocity vector fields computed with omnipolar technology (OT) showing activation originating from the pacing site (average velocity was 40.1 cm/s). **C**, Isochrone map obtained during the same electric stimulation conditions of the same heart, at the same region as in **A** with 10 μmol/L flecanide. The activation pattern is unchanged except for significantly longer total activation time (90 ms). **D**, Corresponding vector field showing similar activation pattern with marked reduced conduction velocity (average velocity: 16.8 cm/s).

We tested the ability of omnipolar electrogram-derived vector fields to detect such changes in the same region of the same heart before and after intervention to reduce CV. Figure 8 shows the effect of flecainide on CV during pacing in an isolated rabbit heart model. Figure 8A and 8B shows an isochrone map and omnipolar vector field generated during 4 Hz pacing (pacing site located on the right) with 1:1 capture. Velocity scale is represented by the color assigned to each vector. After 10 μmol/L flecainide, the isochrone map and omnipolar vector field revealed similar pattern of activation with reduced CV as shown by increased total activation time and lowered velocity vectors detected by the omnipolar electrograms (Figure 8C and 8D).

### Application of Omnipoles in Detecting Multiple Wavefronts

**Figure 9.**
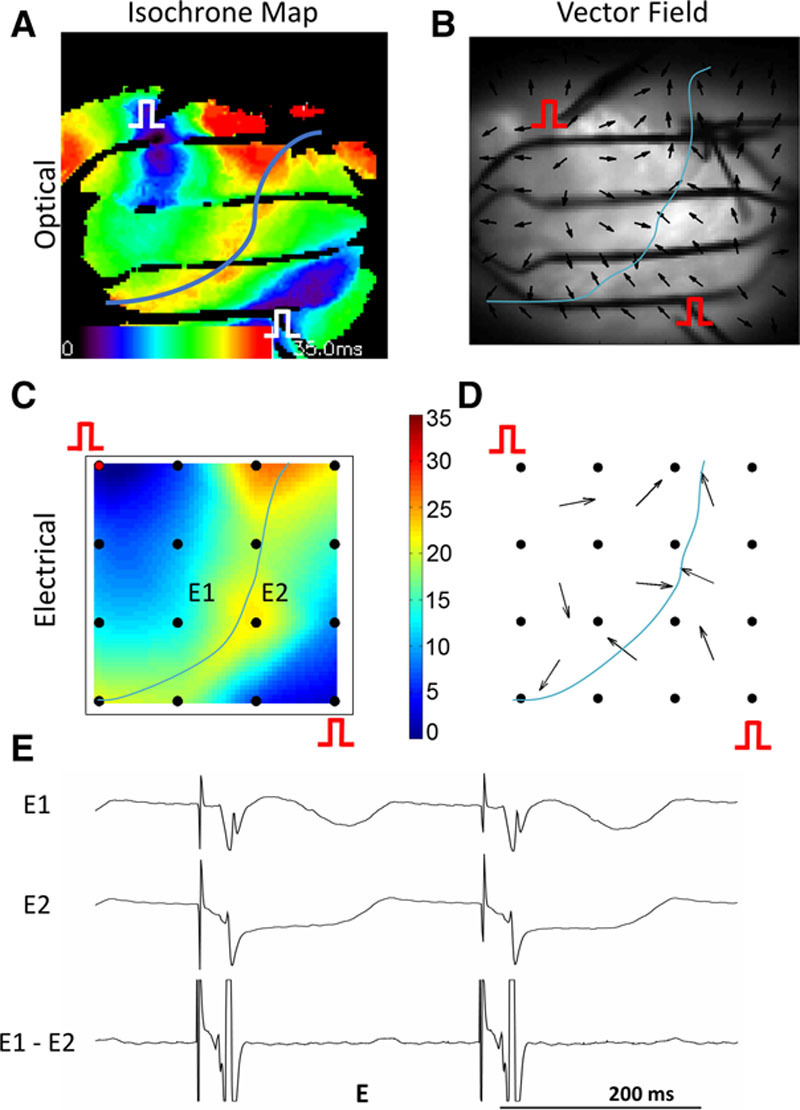
Effect of wave collision on velocity vector field. **A**, Isochrone map obtained from optical data while simultaneous electric stimulation is applied on sites “A” and “B.” **B**, Vector field derived from optical signal revealing uniform centrifugal activation patterns near respective stimulation sites with arrow bumping head to head or bent near collision area shown as a blue line. **C**, Isochrone map obtained from electric data also showing an area of collision in between stimulation sites. **D**, Vector field derived from electric signal also revealing vectors colliding or bending abruptly near collision area (blue line). **E**, Illustrative example of altered unipolar and bipolar electrograms near collision area (shown in **C** as E1 and E2).

The ability of omnipolar electrogram in evaluating 2 separate waves on the same mapping surface was evaluated. Figure 9 shows an example of omnipolar mapping applied during controlled wave collision created by simultaneously pacing sites “A” and “B” on the rabbit model used above. Both isochrone maps (Figure 9A and 9C) clearly show late activity in the middle of the field with a line of collision shown with a blue line. From the vector field, it is possible to locate the line of collision by identifying arrow heads of velocity vector colliding (Figure 9B and 9D).

### Application of Omnipoles to Fractionated Electrograms in Myopathic Human Heart

**Figure 10.**
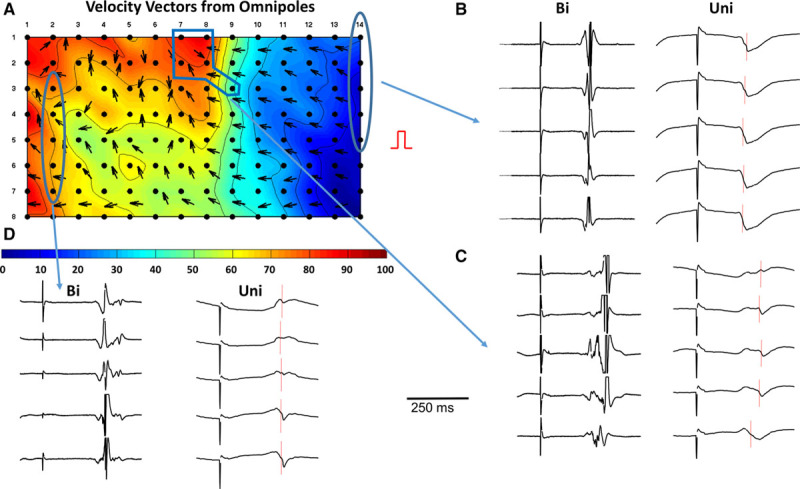
Effect of fractionation and diseased myocardium on velocity vector field. **A**, An isochrone map obtained from electrograms recorded from a high-resolution plaque on a myopathic human heart Langendorff preparation during programmed stimulation. The stimulation site is located on the right side of the plaque. The plaque is composed of 112 electrodes organized as 8 rows by 14 columns, which are labeled on top and on the left of the plaque. Color scale indicates time of activation (in ms) and each isochrone line is spaced by 10 ms. The right side of the map shows relatively ordered iso lines along with uniform velocity vector field, whereas the left side reveals a more complex isochrone map, with a wave colliding from the **top left** and corresponding omnipolar velocity vector disarray. **B** to **D**, The unipolar (Uni) and bipolar (Bi) electrograms associated with 3 areas from the plaque. Increased bipolar electrogram complexity and fractionated potentials are observed from right to left. This corresponds to wave turning and collision from invading wavefront from the **top left**, evidenced both by isochronal color map and overlayed omnipolar vector field.

We used this preparation as we have previously published in this ex vivo model of myopathic human hearts that demonstrate electrograms that show fractionation.^26^ An example showing the omnipolar concept–derived vector field is shown in Figure 10 These data were obtained during pacing. In Figure 10A, an omnipolar vector field overlayed on an isochrone map obtained from a high-resolution plaque electrode array deployed on the endocardial surface of human heart Langendorff preparation. Details on the plaque array are provided in the figure legend. An activation wave is generated and traverses the plaque from right to left. As the wave enters the array, the vector field is organized with associated simple isochrone lines. When the wave reaches column 8, it breaks out and collides with another wave coming from the upper left with associated vector disarray. The fractionated bipolar electrograms correlated with isochronal activation and revealed wave fractionation and collision. More importantly this could be inferred from the local vector field while comparing to the rest of the vector field with uniform conduction. In this way by using the negative image of uniform velocity vector to nonuniform vector field, one could potentially determine areas of wave collision and fractionation, although this will need further extensive validation.

### Application of Divergence to Omnipolar Mapping

**Figure 11.**
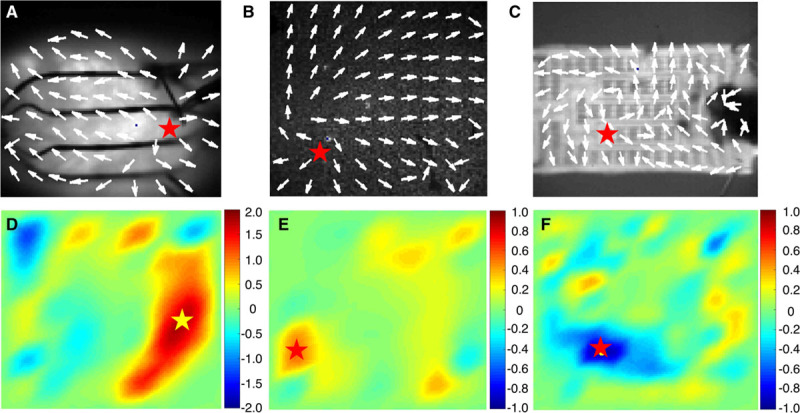
Application of vector fields in determining source locations. we show omnipole-derived vector fields from optically mapped rabbit heart (**A**), cardiac monolayer (**B**), and myocardial construct (**C**) as shown previously; the application of mathematical transforms—divergence and curl—shows the use of omnipolar mapping in determining the exact locations of activation foci (**D**). Moreover, vector fields can portray the vorticity of a rotor or re-entrant circuits whether they are clockwise (**E**) or counter clockwise (**F**).

To date, we have qualitatively determined the locations of apparent sources by looking at the general profiles of vector fields derived with omnipolar electrograms. Unless a known reference is used (eg, pacing electrode location), such a method is highly subjective. By using divergence and curl, we can create maps to quantitatively assess the exact locations of apparent sources within vector fields. An example of a divergence map of a rabbit heart is shown in Figure 11D. Highly positive divergence values were identified on the right side of the map, which is the area near the previously determined location of a pacing electrode (Figure 11A). From its peak, the apparent source profile within a divergence map expands further downward that provides an idea of the extent of divergence’s capability to provide a picture of the direction of a wavefront from an apparent source. In addition, as we move further away from the source, the divergence map shows near zero divergence values, which can indicate uniform wave propagation. Finally, areas with negative divergence values indicate points of convergence for vectors within that area, which could potentially describe exit or collision sites.

### Application of Curl to Omnipolar Mapping

Examples of curl maps obtained from the omnipolar-derived vector fields of cardiomyocyte monolayer and construct are shown in Figure 11E and 11F, respectively, in which strongly positive and negative curl values were calculated near the location of their previously determined rotor core sites (Figure 11B and 11C). As mentioned above, curl maps can also quantify the direction of rotation of the rotors within the substrates, with highly positive values for clockwise rotating sources and extremely negative values for counter clockwise rotating sources. Similar with the divergence maps, curl maps also suggest areas with uniform wave propagation, where curl values are near zero with respect to the curl values of an apparent source.

### Evaluation of Potential Clinical Performance: Electric Mapping Validation

Although omnipolar methodology applied to optical-mapping data has shown promise in tracking sources and AD in vitro, optical mapping is not clinically used and validation has to relate to traditional electric mapping. To evaluate the potential use of omnipolar electrograms within a clinical setting, we validated omnipolar electrogram–derived mapping against data we collected from traditional electrograms from the 16-electrode array. The unique concurrent set up with traditional electrograms on a rabbit Langendorff allowed also for creation of 3×3 omnipolar vector field profiles (Figure IIA in the Data Supplement), which were interpolated to 9×9 grids (Figure IIB in the Data Supplement) for ease of visualization. These grids were then used to perform a blind study involving 4 electrophysiologists who were asked to determine the approximate area of focal source, if it existed. On average, electrophysiologists located apparent focal sources 2.77±2.22 mm away from activation foci, which were previously determined from the earliest time point from corresponding isochronal maps (Figure IIC in the Data Supplement). Furthermore, electrophysiologists have located these apparent focal sources 3.03±2.82 mm away from previously determined locations of activation foci on optically mapped hearts.

## Discussion

This study elucidates and validates the concept that local myocardial AD and speed can be derived without the tedious process of creating LAT maps, which requires meticulous identification of activation times by standard techniques. This paradigm obviates the limitations created by bipolar electrode orientation with respect to an approaching wavefront. We validated such a technique against a robust reference by evaluating their agreement with a traditional methodology. Finally, we have shown that omnipolar electrograms provide consistent and predictable results in an in vivo context compared with their unipole and bipole counterparts. In this study, we demonstrated that cardiac mapping using omnipolar electrograms allows for generation of an accurate velocity vector field. Subsequent application of known mathematical methods, such as divergence and curl, to this vector field can be used for detection of focal cardiac sources.

The identification of sources of cardiac arrhythmia relies on mapping the depolarization of propagating wavefronts and interpreting the resulting directional information. Crucial to all of these methodologies is the determination of local arrival time. Although typically LAT is based on the instant of maximal rate of voltage change, exceeding a threshold, or subjective assignment, alternate strategies to LAT assigned isochrones have been explored: Weber et al^8^ used circular mapping catheters and a cosine method, whereas Ideker et al^7^ used fitting polynomial functions as an approach to determine arrival times and analytically obtaining CV vectors; Dubois et al^9^ used a multispline catheter for planar fit technique for generating CV vectors from LATs; and Fitzgerald et al^27^ and Shors et al^28^ used Hilbert transforms and cross correlations to improve the estimation of arrival times and also fit CV vectors to the resulting set of LATs. Although it may be argued that this is indeed a more robust way to assign a LAT than traditional *dv*/*dt*-based methods, this approach has its own practical limitation. At the time the Hilbert transform–based approach was proposed, even if the appropriate waveform was analyzed, it was computationally impractical because of the lack of available computing power and the cumbersome nature of the operation. At the end, the issue of time and space resolution for building accurate CV vector fields still remains unsolved and is unlikely ever to be in a clinical context where high-resolution arrays are rarely used.

Another alternative strategy was pioneered by Kadish et al^5^ using a vector electrogram-based approach. Omnipole shares common concept of reliance on multielectrode catheters and single beat, local processing and improves on Kadish et al’s^5^ method by providing velocity amplitude in addition to direction. More importantly, the vectorial approach by Kadish et al^5^ and the approach by Fitzgerald et al^27^ have not been incorporated into 3D electro anatomic mapping for practical mapping work clinically because of multiple factors, the most important being computationally expensive at the time they were introduced to be implemented. Omnipolar electrograms as demonstrated here, readily provides an observer the orientation of a wavefront as it uses less resource in the process. Therefore, omnipolar electrograms together with previously described quantitative methods provide a practical basis for determining local AD of wavefronts on the myocardium, which can then be appropriately incorporated in 3D electroanatomic mapping systems and could be widely used in cardiac arrhythmia mapping.

Our analysis suggests that divergence and curl can reveal locations of focal and rotary sources within a given media. As expected, this source detection technique was more effective when the sources within the area interrogated by the mapping tool. For cases in which the sources were readily identifiable, such as the location of the pacing electrode on a rabbit heart in the case of our optical maps, divergence algorithm performed well as sources were positioned within the CMOS camera’s field of view. However, we observed with our monolayers that sources in most plates were usually outside of the MEA’s field of view and, thus, were not readily identifiable. Consequently, omnipole-derived vector fields that do not contain rotors or sources within the field of view can be used to backtrack to a source based on the orientation of the vectors. Only until then can divergence and curl of a vector field be calculated to pinpoint the exact position of a source. This could be useful, for example, in localizing potential ablation sites.

In catheterization electrophysiology laboratory, it is today challenging to pinpoint the exact location of a source unless sufficient spatial and temporal data have been collected. Recent advances have led to rapid automated annotation by cardiac mapping system. These approaches still require the completion of the mapping process to localize activation source. A process that allows this localization during mapping would be a paradigm shift. The real-time feedback at the catheter tip with beat-to-beat directional information could open a new chapter in cardiac mapping. Clinical operators could take advantage of not having to complete cardiac chamber map but interactively use the wavefront information in the mapping tool to map toward the source of arrhythmia. Therefore, it is important to evaluate the use of omnipolar mapping as a tool in real time in a drag-and-map mode that would allow for identifying cardiac sources, and this will be validated prospectively in the clinical arena. This study provides the basis for the use of specialized catheters and novel signal processing techniques to determine and visualize CV and AD from an omnipolar electrogram for a single local depolarization.

### Limitations

The studies described here were conducted in controlled conditions from cell cultures, tissue, and structurally normal hearts. It is unknown if omnipolar electrogram–derived data on heterogeneous myocardial surface and curved wavefronts within the clique of electrodes providing omnipolar electrograms will perform with practical use and will need investigation. Because a travelling wave is required for measuring velocity, omnipolar electrograms could not be used over focal points (breakthroughs) because velocity could not be measured with any methods. This is particularly true for epicardial velocity measurements during sinus rhythm. Although omnipolar electrograms will provide accurate physiology of single travelling wave, the effects of wave collision and fractionation deserve additional validation work, especially omnipolar analysis of fibrillatory signals from the clinical arena is unknown. It is also intriguing to consider the depth of myocardium and its contribution to the omnipolar electrogram with obvious implication to transmural mapping. Additional refinements such as optimal effect of electrode size and resolution on omnipolar-derived variables such as speed, direction, and amplitude are needed, although the performance with tested resolution and size was in agreement with reference standards.

### Conclusions

Omnipolar electrogram methodology provides a novel catheter electrode orientation- and activation timing independent means to characterize wavefront propagation physiology, with speed and direction, for each cardiac cycle. When omnipolar-derived data are combined with mathematical operators curl and divergence, it allows for efficient detection of cardiac sources that could be incorporated in electroanatomic mapping systems for potential clinical use. This methodology will need careful clinical validation to determine practical therapeutic use.

## Acknowledgments

We thank Drs Wang and Gramolini for their contributions in the creation of cardiac monolayers; Mr Lai, Drs P. Lee, and M.A. Azam for their assistance in the setup and execution of our whole rabbit heart Langendorff protocol; Dr Leslie Loew for providing the near infrared voltage dye DI-4-ANBDQPQ; and Mr Veluppillai for article editing.

## Sources of Funding

This study was funded by St. Jude Medical, St. Paul, MN.

## Disclosures

At the time the studies were conducted and this article was drafted, S. Massé and Dr Nanthakumar were research consultants for St. Jude Medical. Drs Balachandran and Deno were both employees of St. Jude Medical. The other authors report no conflicts.

## Supplementary Material
