# Supplementary material for: Resolving Myocardial Activation With Novel Omnipolar Electrograms
Source: Circ Arrhythm Electrophysiol. 2016 Jul 19;9(7):e004107. doi: 10.1161/CIRCEP.116.004107 (PMC4956680; doi:10.1161/CIRCEP.116.004107)
Supplement: Supplementary file 1 [file hae-9-e004107-s001.pdf]

Supplemental Material

Supplemental Methods

Neonatal Mice Cardiomyocytes Monolayer Preparations

Cardiac monolayer cells were prepared following the procedures found in (1). Briefly, ventricles from 2 day-old C57/BL6 mice were dissociated enzymatically with collagenase II 1mg/ml (Worthington) at room temperature. Myocytes were collected following 3 hours of digestion and were pooled to be re-suspended in growth medium: DMEM/ F12 (ThermoFisher Scientific) supplemented with 10% fetal calf serum, 10% horse serum, 100 U/ml penicillin, 100 mg/ml streptomycin (ThermoFisher Scientific). To reduce fibroblast content, cell suspensions were pre-plated on culture dishes for 1 hour at 37˚C with 5 mg/100ml bromodeoxyuridine (BrdU; Sigma). Subsequently, myocytes were seeded on 1.4 cm by 1.4 cm 64-channel microelectrode array (MEA) plates, pre-coated with Matrigel (ECM Gel from Engelbreth-Holm-Swarm murine sarcoma, Sigma, E1270) diluted in ddH2O with a ratio of 1:10. The cells had an approximate density of $2x10^{7}$ cell/ml. 25 µl cell suspension (approximately 4.0 $x10^{5}$ – 5.0 $x10^{5}$ cells) were directly plated onto each MEA plates. The cells were then cultured in a 5% CO2 + 95% air, at 37°C incubator for 24 hours. Unsettled cells were removed by swirling fresh medium. Spontaneous activity was recorded using optical and electrical methods described in the manuscript.

Three Dimensional Myocardial Constructs derived from Induced Pluripotent Human Stem Cells

Myocardial tissue constructs were created for this study from human IPS according to the following procedure: a biodegradable, UV-polymerizable, elastomer, poly(octamethylene maleate (anhydride) citrate) (2, 3) was used for building tissue scaffolds. A 3D cardiac tissue construct was created using a novel microfludic scaffold that more faithfully reproduced architectural complexity of the native tissue. Specifically, the scaffold contains perfusable inner lumens that were fully endothelialized to mimic the vasculature. In the parenchymal space, the scaffold lattice was fully interconnected to allow the cardiomyocytes to connect to one another in the entire space (x,y, and z-direction) thus mimicking the cardiac syncytium. The scaffolds were incubated for 2 hours after which 4 mL of endothelial cell (EC) media was added while increasing the flow rate of perfusion. The endothelial cells were allowed to proliferate overnight, forming a confluent network of cells. The techniques described by (4) for harvesting and culturing HES-3 NKX2-5 GFP positive cells were employed in the creation of in-vitro cardiomyocytes. Cultured cardiomyocytes mixed with 15 µL Matrigel (BD Biosciences) at 100 x 10^{6} to 200 x 10^{6} cells/mL were seeded in to endothelialize scaffolds. Gelation of the cell media was allowed for 30 minutes at 37°C. After seeding, 4 mL of cardiomyocyte media was added, increasing perfusion rate.

Validation of CV measurements with OT – Rabbit Langendorff Model

Briefly, a least square method was used to fit a surface over the timing data of a 4-electrode group and a CV vector were obtained using the following equations, similar to the ones used by Bayly (5):

$$T(x, y) = axy + bx + cy + d$$

$$T_x = \frac{\partial T}{\partial x}, T_y = \frac{\partial T}{\partial y}$$

$$V_c = \begin{bmatrix} \frac{dx}{dT} \\ \frac{dy}{dT} \end{bmatrix} = \begin{bmatrix} \frac{T_x}{T_x^2 + T_y^2} \\ \frac{T_y}{T_x^2 + T_y^2} \end{bmatrix}$$

This was repeated for all contiguous groups of 4 electrodes to obtain a 9 x 9 CV vector field.

Concurrent Mapping Protocol – Electrical and Optical Mapping

Simultaneous unipolar and bipolar electrograms were recorded while optical images were obtained as described in the supplementary methods (sampling rate unipolar: 1 kHz, bipolar: 2 kHz) and band-pass filtered (unipolar: 0.5 Hz – 200 Hz, bipolar: 28 Hz – 750 Hz).

As the heart was perfused, a 20 µL bolus of 27 mmol/L (in pure ethanol) near infrared voltage dye, DI-4-ANBDQPQ, was injected without recirculation as a single bolus in to the coronary circulation for about five minutes (6, 7). The heart was mechanically decoupled by injecting 1.25 mg of Blebbistatin (Enzo Life Sciences, Inc.) added to approximately 1 L of re-circulating perfusion solution through an access on the bubble trap and was electrically paced from 4 pre-determined different sites at 3 Hz, with a 0.5 ms pulse width, using the stimulator described above. The 4 sites were chosen as follow: Sites A & B were chosen on each longitudinal side of the HD Grid™; site C was located near the base of the left ventricle and site D was in the middle of the square formed by the HD Grid™. The fluorescence was excited at 640 nm using an ultra-bright LED (Luminous Devices) coupled to a filter (Chroma D640X/20X). Emission was captured through a Navitar lens and a 700 nm long pass filter and recorded with our optical mapping system as described above. Frame rate was 3333 frames/s and the field of view was 2.4 cm^2 (0.24 mm/pixel).

Divergence and Curl

Let a two dimensional vector field over the same myocardial surface be denoted by $\mathbf{V}(x,y) = A(x,y)\hat{\mathbf{x}} + B(x,y)\hat{\mathbf{y}}$, divergence is a vector operator which provides the magnitude of sources and sinks in terms of a scalar. Mathematically, we may estimate the 2-D divergence (div) at a location (x_1, y_1) near (x_2, y_2) using a discrete approximation to differentiation

$$\mathrm{div}(V) = \nabla \cdot \mathbf{V} = \frac{\partial A}{\partial x} + \frac{\partial B}{\partial y} \cong \frac{\Delta A}{\Delta x} + \frac{\Delta B}{\Delta y} = \frac{A(x_2, y_1) - A(x_1, y_1)}{x_2 - x_1} + \frac{B(x_1, y_2) - A(x_1, y_1)}{y_2 - y_1}$$

The result of the equation above is a scalar value that describes the flow of a vector field at a particular area: positive values indicate that the flow of the field behaves like a source from which closely spaced vectors radiate outwards. Conversely, negative values indicate that the flow of the field behaves like a sink and is described with a group of vectors that converge in to a specific point.

The curl of 2-D vector field quantifies a characteristic of a spiral called vorticity which determines the direction and magnitude of vector rotation. Curl within $\mathbf{V}(x, y)$ may be similarly expressed as follows

$$\mathrm{curl}(V) = \nabla \times \mathbf{V} = \left(\frac{\partial B}{\partial x} - \frac{\partial A}{\partial y}\right)\mathbf{i} = \frac{B(x_2, y_1) - B(x_1, y_1)}{x_2 - x_1} - \frac{A(x_1, y_2) - A(x_1, y_1)}{y_2 - y_1}$$

The equation above yields a scalar which describes the orientation of a rotation at specific points throughout the vector field: positive values pertain to a counter clockwise rotating source while negative values pertain to a clockwise rotating source. For all models, we used cubic interpolation (8) to fit the div and curl maps to the size of the original media so that we can obtain a comparable spatial scale and, in turn, sufficient registration.

We determined the values that div and curl yield in ideal conditions, where a source is placed in the middle of a vector field, by creating four 7-by-7 vector field patterns to simulate a focal source, a focal sink, a clockwise (CW) rotary source, and a counterclockwise (CCW) rotary source, as shown in Supplementary Figure 3. We then calculated the div maps for both the focal source and focal sink patterns and the curl maps for both the CW and CCW rotary source patterns. We determined ±2 to be the upper and lower boundary values for divergence maps while ±1 are the upper and lower boundary values for curl maps. Such boundary values were used to limit the div and curl maps' color scale to their maximum and minimum values.

Supplemental Figures

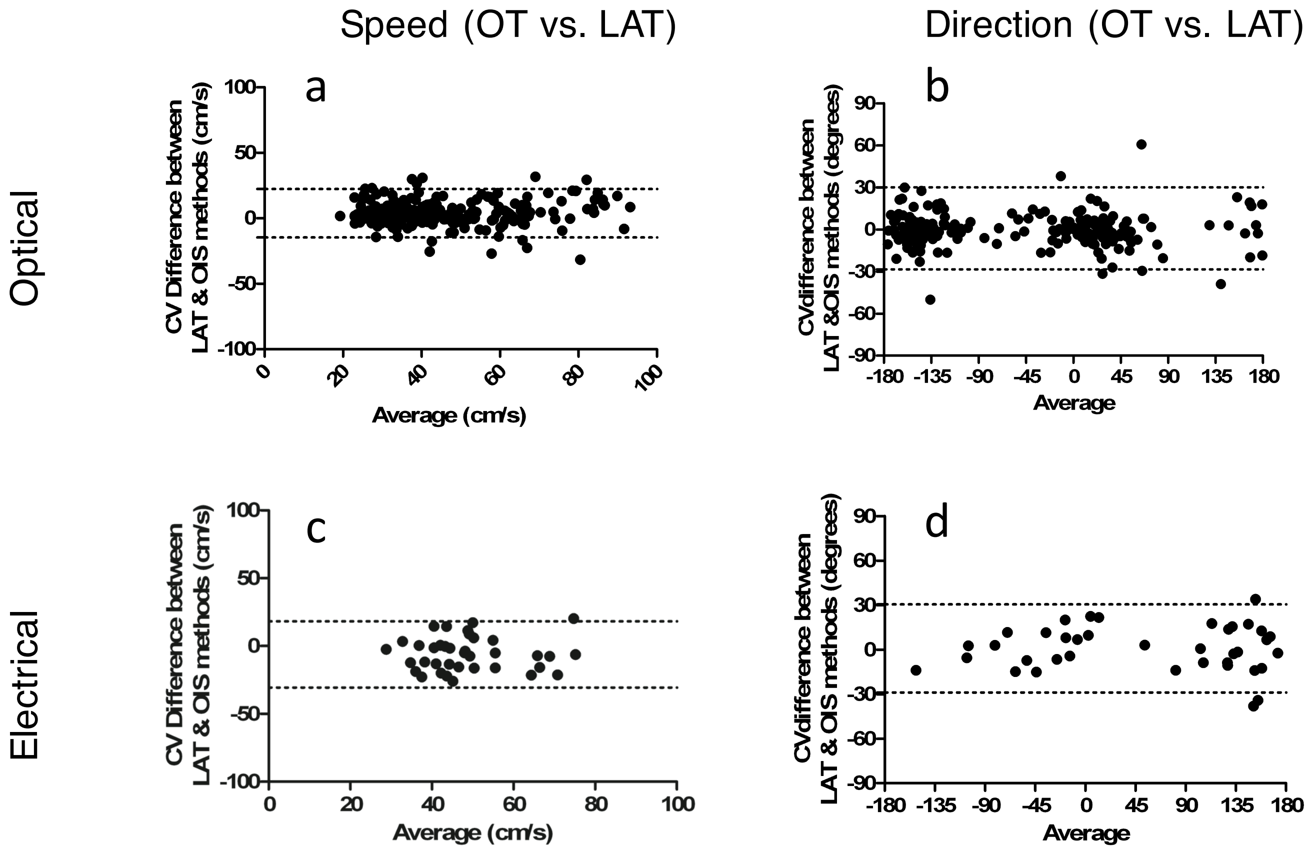

Supplement Figure 1: Bland-Altman plots of comparing variations in speeds and directions between omnipolar (OT) and LAT methods: here we show the agreement between the OT and LAT methods of calculating conduction speeds from optically- (**a**) and electrically-mapped (**c**) data; we also show such an agreement between in their conduction directions from optically- (**b**) and electrically-mapped (**d**) data. Dashed lines denote the 95% confidence intervals

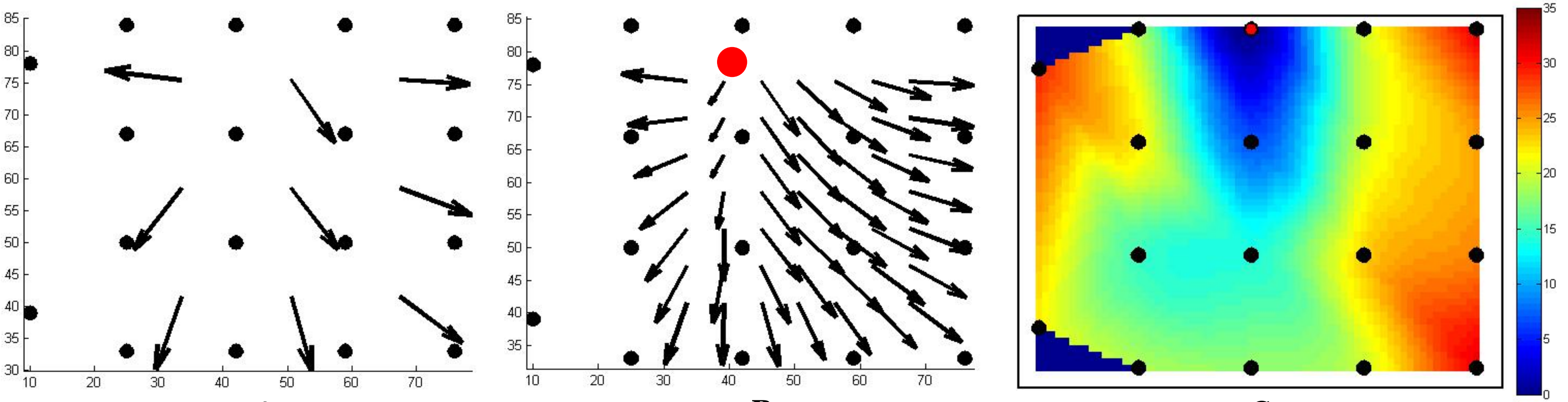

A **B** **C**

Supplement Figure 2: A sample progression of interpolation of a 3-by-3 OIS vector field (left) to a 7-by-7 OIS vector field (middle) with the position of the approximate location of an associated source for an isochrone map all measured in milliseconds (right)

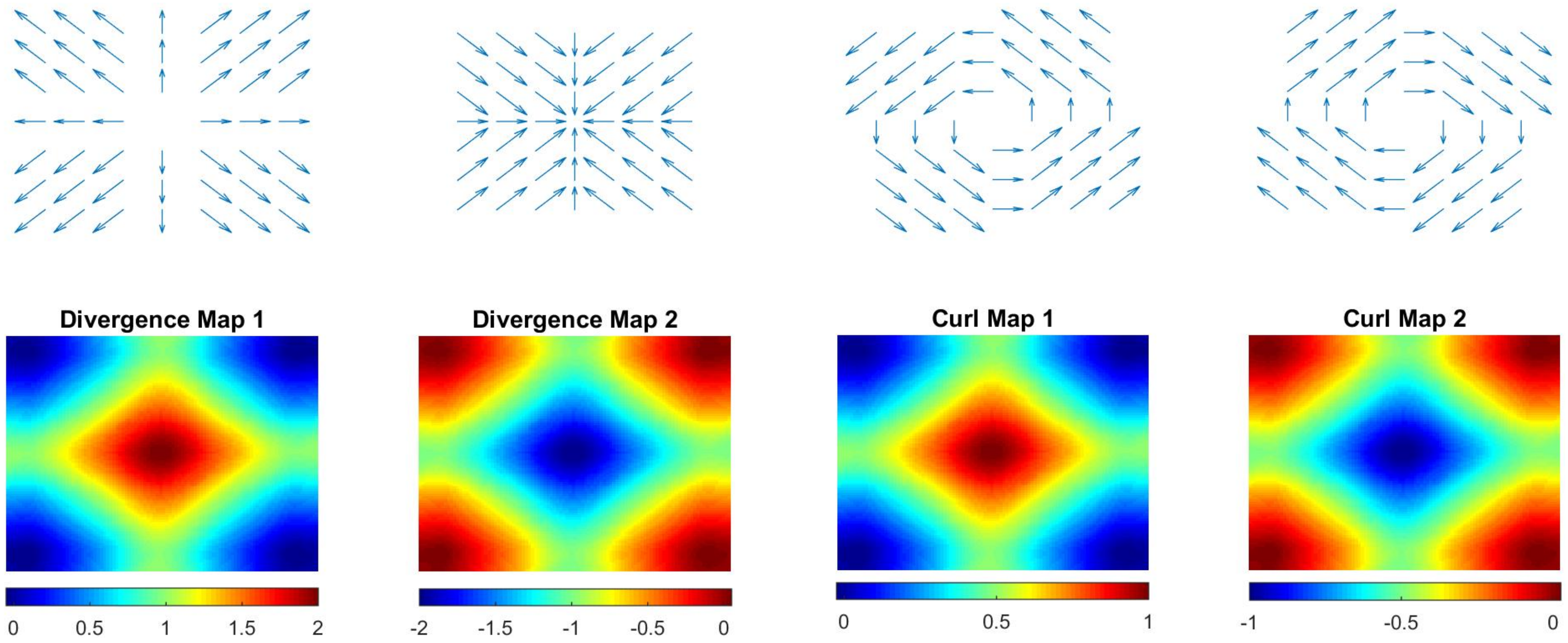

Supplement Figure 3: Divergence and Curl applied to synthetic vector fields to verify its function in detecting focal and rotary sources; div maps 1 and 2 illustrate examples of how a source and a sink is represented from an OT vector field, respectively; in addition, curl maps 1 and 2 illustrate examples of how a clockwise and a counter-clockwise rotations within an OT vector field, respectively, are represented.

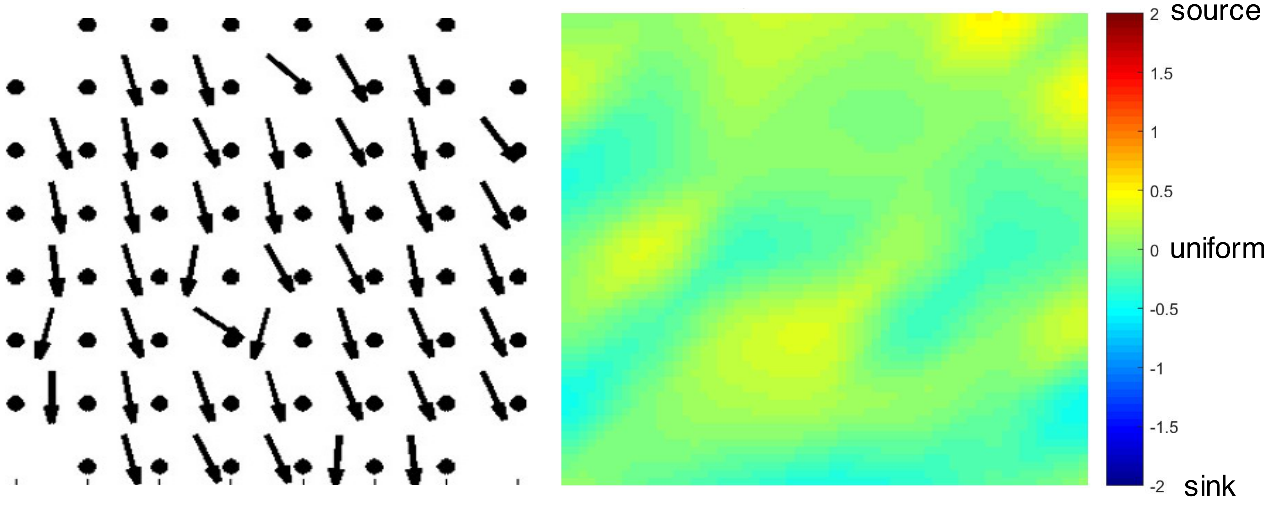

Supplement Figure 4: Application of divergence on uniformly propagating waves in an cardiac monolayer; as in supplement figure 1, a div value of 2 indicates the presence of a source, a div value of 0 indicates a uniform propagation, and a div value of -2 indicates the presence of a sink

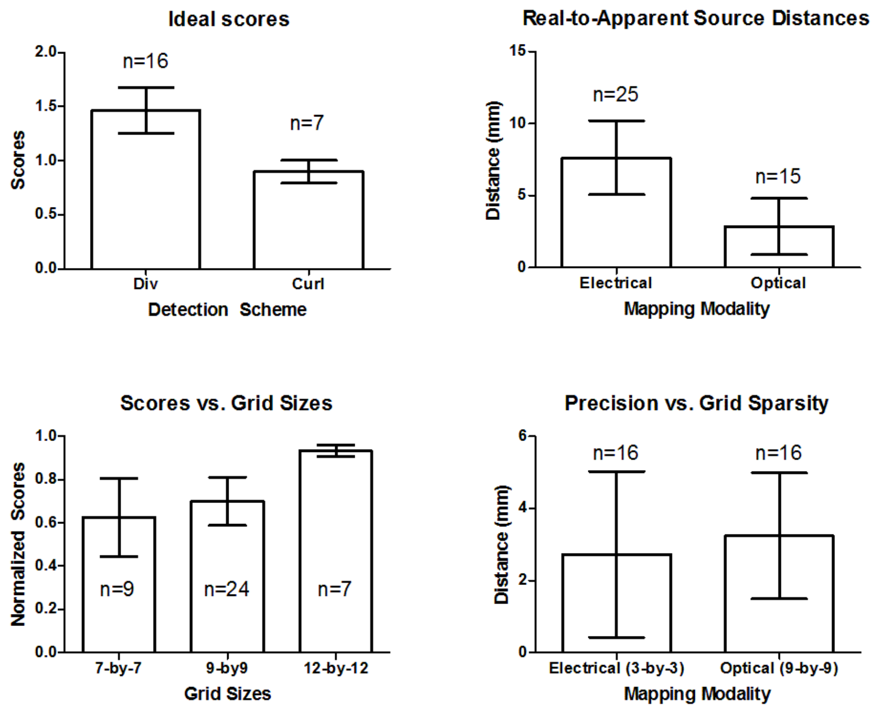

Supplement Figure 5: Range of ideal div and curl scores used hand-in-hand with ideal real-to-apparent source distance to establish a threshold for source detection (**top left**); distance and distance variations of real-to-apparent source distance with respect to the mapping modality used i.e. electrical and optical mapping (**top right**); score and score variations in relation to the size of grids used to calculate divergence and curl (**bottom left**); and precision (standard deviation) of source detection in terms of distance in millimeters in whole rabbit hearts using our concurrent mapping setup (**bottom right**).

References

1) Wang DY, Abbasi C, El-Rass S, Li JY, Dawood F, Naito K, Gramolini AO. Endoplasmic reticulum resident protein 44 (ERp44) deficiency in mice and zebrafish leads to cardiac developmental and functional defects. *Journal of the American Heart Association*. 2014; 3(5): e001018.

2) Tran RT, Thevenot P, Gyawali D, Chiao JC, Tang L, Yang J. Synthesis and characterization of a biodegradable elastomer featuring a dual crosslinking mechanism. *Soft Matter*. 2010; 6(11): 2449-2461.

3) Yang J, Webb AR, Ameer, GA. Novel Citric Acid-Based Biodegradable Elastomers for Tissue Engineering. *Advanced Materials*. 2004; 16(6): 511-516.

4) Kennedy M, D'Souza SL, Lynch-Kattman M, Schwantz S, Keller G. Development of the hemangioblast defines the onset of hematopoiesis in human ES cell differentiation cultures. *Blood*. 2007; 109(7): 2679-2687.

5) Bayly PV, KenKnight BH, Rogers JM, Hillsley RE, Ideker RE, Smith WM. Estimation of conduction velocity vector fields from epicardial mapping data. *Biomedical Engineering, IEEE Transactions on*. 1998; 45(5): 563 – 571.

6) Lee P, Yan P, Ewart P, Kohl P, Loew LM, Bollensdorff C. Simultaneous measurement and modulation of multiple physiological parameters in the isolated heart using optical techniques. *Pflügers Archiv-European Journal of Physiology*. 2012; 464(4): 403-414.

7) Lee P, Bollensdorff C, Quinn TA, Wuskell JP, Loew LM, Kohl P. Single-sensor system for spatially resolved, continuous, and multiparametric optical mapping of cardiac tissue. *Heart Rhythm*. 2011; 8(9): 1482-1491.

8) Amidror, I. (2002). Scattered data interpolation methods for electronic imaging systems: a survey. *Journal of Electronic Imaging*, 11(2), 157-176.

Legends for the video files

Video file #1: Flashing dot display showing the spontaneous sequence of activation of a monolayer cell culture on a micro electrode array (MEA). Each red dot represents an electrode from the MEA that is lit when the dv/dt of the recorded unipolar electrogram exceeds a set threshold. The video shows a planar wave that moves across MEA from the upper left to bottom right of the MEA.

Video file #2: Optical mapping of a monolayer cell culture showing a rotor that is anchored in the lower left corner. Wavebreaks can be seen away from the rotor core at the bottom right.

Video file #3: Optical mapping of a 3-D engineered tissue construct. This video shows corresponding wavefronts generated from two counter-rotating rotors spontaneously present during optical mapping. The darker area on the left is a shadow from a pacing electrode.

Video file #4: Optical mapping of rabbit Langendorff during OT validation. This video shows a wave generated while pacing from site A (located in the bottom right). The heart orientation on this video and figures is: Apex on left, base on right. The view is from LV anterior. The dark areas represent the HD Grid™ and some of the electrodes used for pacing. The activation sequence does not radiate from the pacing electrode due to tissue anisotropy.

Video file #5: Pre-clinical implementation of OT in 3D electro-anatomical mapping. Two OT-enabled catheter are shown during a live-session of mapping on a research version of EnSite Velocity™ system in an in-vivo swine EP study. Here the HD Grid™ was positioned on superior LA septum (left panel) while the S120™ was located in mid RA (mid panel). The initial video segment shows velocity vectors produced during sinus rhythm, followed by an abrupt change caused by stimulating from the lower RA.

Video file #6: OT “drag-and-map”. This example shows how one would map a source using OT catheter. For demonstration purposes, the sinus node is used as a source of cardiac activation. The velocity vectors are changing respective to the Sinus Node on a beat-by-beat basis while the HD Grid™ is moved in the right atria. This change on velocity vectors could be used to track a source upstream until a starburst indicative of a source is obtained.
